# Prevalence and Genotyping of *Mycobacterium avium* subsp. *paratuberculosis* in Sheep from Inner Mongolia, China

**DOI:** 10.3390/vetsci12040326

**Published:** 2025-04-02

**Authors:** Rong Zhang, Yue-Rong Lv, Bo Yang, Hao Wang, Jun-Tao Jia, Zhi-Hong Wu, Ming Nie, Lian-Yang Sun, Shi-Yuan Xue, Yu-Lin Ding, Rui-Bin Chen, Siqin Tunala, Li Zhao, Yong-Hong Liu

**Affiliations:** 1 Otok Banner Animal Disease Prevention and Control Center, Ordos 016100, China; zr_129@163.com (R.Z.); cyb_0921@163.com (R.-B.C.); nmhfei@126.com (S.T.); 2 College of Veterinary Medicine, Inner Mongolia Agricultural University, Hohhot 010018, China; lyr765619420@163.com (Y.-R.L.); wh1042479310@163.com (H.W.); xsy55145600@163.com (S.-Y.X.); dingyulin2001@126.com (Y.-L.D.); 3 Animal Disease Control Center of Ordos, Ordos 017000, China; 15886840959@126.com; 4 Vocational and Technical College, Inner Mongolia Agricultural University, Baotou 014109, China; juntaojia1982@imau.edu.can; 5 Agriculture and Animal Husbandry Technology Popularization Center of Inner Mongolia Autonomous Region, Hohhot 010010, China; wzh8410051@163.com; 6 Alxa Left Banner Animal Disease Prevention and Control Center, Alxa Left Banner 750300, China; nieming0314@163.com; 7 Zhalantun Animal Disease Prevention and Control Center, Zhalantun 162650, China; 18748237178@163.com

**Keywords:** *Mycobacterium avium* subsp. *paratuberculosis*, qPCR, prevalence, genotype, China

## Abstract

Background: Paratuberculosis (PTB) is a chronic wasting disease mainly caused by *Mycobacterium avium* subsp. *paratuberculosis* (MAP) in ruminants. It is difficult to diagnose, prevent, treat, and eradicate, thereby causing serious economic losses to the livestock industry. Therefore, finding a detection method with high sensitivity and specificity is crucial to preventing and controlling PTB. Methods: A total of 1585 fresh fecal samples were collected from 12 prefectures and cities across Inner Mongolia between March 2022 and October 2024. The samples were subjected to pretreatment, followed by DNA extraction. Subsequently, MAP detection and genotyping were performed using a two-step qPCR method. Results: The overall prevalence of MAP in ovines was 3.34% (53/1585), with the prevalence in 12 prefectures and cities ranging from 0% (0/100) to 7.73% (15/194). In the eastern, central, and western regions, the prevalence rates were 4.74% (31/654), 3.68% (14/394), and 1.49% (8/537); in small-scale and intensive farms, they were 3.23% (22/682), and 3.56% (31/903); and in goats and sheep, they were 0.91% (2/219) and 4.98% (36/723), respectively. The overall prevalence rates of C- and S-type MAP were 2.90% (46/1585) and 0.44% (7/1585), respectively. Conclusions: To the best of our knowledge, this study is the first to conduct an epidemiological investigation of PTB in sheep across all nine cities and three leagues in Inner Mongolia and to perform MAP typing on a large scale. It elucidated the differences in the prevalence of PTB in different regions of Inner Mongolia and found that geographical location and sheep breed are potential risk factors for the differences in MAP prevalence. Furthermore, it has been shown that C- and S-type MAP coexist in the eastern and central regions of Inner Mongolia.

## Simple Summary

In this study, a total of 1585 fresh fecal samples were collected from Inner Mongolia. DNA extraction from the samples was performed, followed by MAP detection and genotyping. The overall prevalence of MAP in ovines was 3.34% (53/1585). The overall prevalence rates of C- and S-type MAP were 2.90% (46/1585) and 0.44% (7/1585), respectively. To the best of our knowledge, this study is the first to conduct an epidemiological investigation of PTB in sheep across all nine cities and three leagues in Inner Mongolia and to perform MAP typing on a large scale.

## 1. Introduction

Paratuberculosis (PTB) is a chronic wasting disease mainly caused by *Mycobacterium avium* subsp. *paratuberculosis* (MAP) in ruminants. It is characterized by a gradually progressive emaciation and increasingly severe diarrhea in affected animals [1]. Considering the substantial impact of PTB on economic stability, animal welfare, and public health, this disease is widely recognized as an important concern [2]. Moreover, a growing body of evidence suggests that MAP is a zoonotic pathogen [3].

Based on the core genome analysis, MAP is classified into two distinct lineages: Type C (Type Ⅱ) and Type S. Type C also includes Type B, which can be further subdivided into the “Indian Bison type” and “USA Bison type”. Type S can be further subdivided into sub-group Types I and Ⅲ and sub-lineages of camelid isolates [4]. PTB is prevalent globally, affecting numerous regions across different continents [5]. Infections of ovine with MAP have been documented in numerous countries, including New Zealand, Australia, and South Africa in the Southern Hemisphere; Norway, Great Britain, and Austria in the Northern Hemisphere; and Mediterranean countries such as Greece, Portugal, Morocco, Spain, and Jordan [6]. Notably, it has seroprevalence rates of 67% in the Republic of Cyprus [7], 76.9% in the USA [8], 74.3% in Chile [9], 62.9% in France [10], 79.4% in India [11], and 44.1% in Argentina [12], while the global herd-level seroprevalence rate was 55.51% [13].

In China, reports on the prevalence of ovine PTB are scarce, and no systematic molecular epidemiological studies have been conducted to date. In Inner Mongolia, a prevalence of 18.48% was determined in Bayannur using serological methods [14], with six cities having a prevalence of 7.60% [15]. This study aimed to leverage the high specificity and sensitivity of qPCR using sequence-specific fluorescently labeled probes [16]. Moreover, a two-step qPCR method was employed to distinguish between Type S and C MAP, enabling a comprehensive investigation of the infection status and genotypes of MAP in sheep across the entire Inner Mongolia region.

## 2. Materials and Methods

### 2.1. Study Areas and Sample Collection

**Figure 1:**
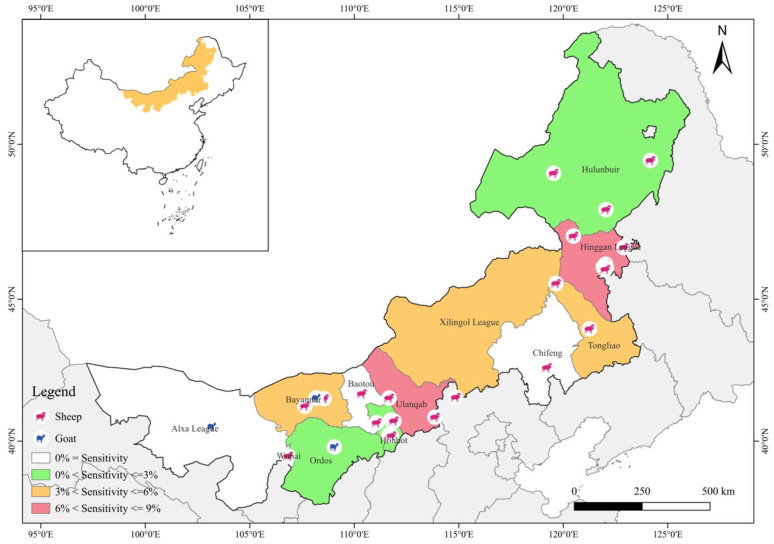
Geographic map of the sampling locations in Inner Mongolia, China. The figure was originally designed by the authors using QGIS 3.42 software. The original vector diagram imported in QGIS was adapted from MAP WORLD (https://www.tianditu.gov.cn/?4) (accessed on 2 February 2025).

From March 2022 to October 2024, a total of 1585 fresh fecal samples (30–50 g each) were rectally collected from 30 sheep farms or households across 9 cities and 3 leagues in Inner Mongolia (Figure 1). The samples were specifically collected from the eastern region (n = 654, 126°04′ E–126°29′ E, 37°24′ N–53°23′ N), central region (n = 394, 111°13′ E–111°78′ E, 40°34′ N–40°58′ N), and western region (n = 537, 97°10′ E–97°78′ E, 37°24′ N–53°23′ N). During the collection process, each sample was assigned a unique identifier, and detailed records were maintained for the collection location, rearing method, and sheep breed. The samples were placed in frozen sampling containers (refrigerated with ice packs), transported to the laboratory within 1–2 days, and stored at 4 °C DNA extraction as soon as possible.

### 2.2. DNA Extraction

After the pretreatment of fecal samples, genomic DNA was extracted using the E.Z.N.A.^®^ Stool DNA Kit (Omega Bio-tek, Norcross, GA, USA) in a biosafety cabinet, strictly adhering to the manufacturer’s protocol. Subsequently, the extracted DNA was stored at −20 °C for future analysis.

### 2.3. qPCR

**Figure 2:**
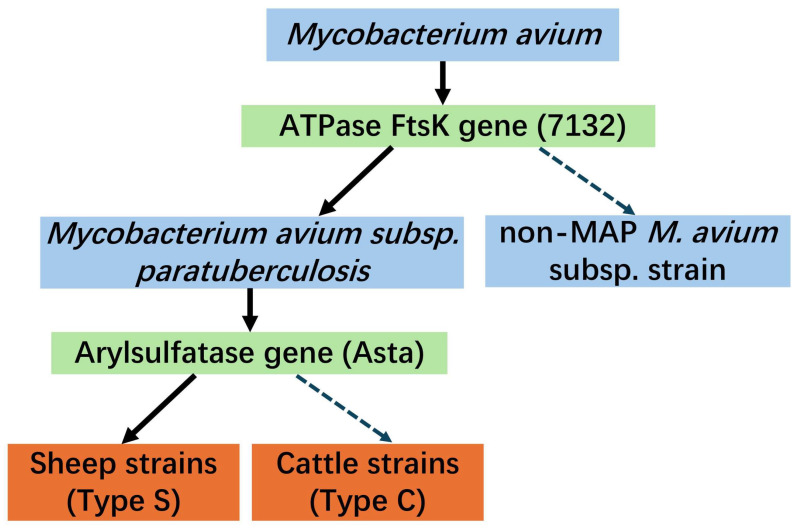
MAP Classification Roadmap.

The Premix Ex Tag™ (Probe qPCR) (TaKaRa, Beijing, China) kit was used to amplify the ATPase FtsK (886451–887722) and Arylsulfatase (1877760–1879361) genes of the DNA (Figure 2). The primers and probes were labeled with FAM at the 5′ end and DBQ1 at the 3′ end [17] and were synthesized by Sangon Biotech (Shanghai, China). Amplification and fluorescent detection were performed on the Applied Biosystems^®^ QuantStudio™ 7 Flex. The FtsK PCR cycling program consisted of an initial denaturation step at 95 °C for 60 s, followed by 45 cycles of denaturation at 95 °C for 15 s and annealing at 60 °C for 30 s. Meanwhile, the arylsulfatase PCR cycling program consisted of an initial denaturation step at 95 °C for 60 s, followed by 35 cycles of denaturation at 95 °C for 15 s and annealing at 65 °C for 30 s. The amplification reaction for all the MAP-specific assays consisted of 20 μL of Premix, 0.2 μM of primers (final concentration), 0.2 μM of probe (final concentration), and 2 μL of template.

The limit of detection (LOD, defined as the lowest dilution where 100% of replicates were positive) for the gene-specific assays of FtsK and Arylsulfatase was used as the criterion for positive results. Specifically, the LOD for FtsK was 0.0002 fg/μL with Cq values ranging from 42.6, while for Arylsulfatase it was 0.004 fg/μL with Cq values ranging from 34.9.

### 2.4. Statistical Analysis

Differences in MAP prevalence among regions, feeding methods, breeds, and genotypes were compared using the chi-square test via SPSS Statistics 26.0 (IBM Corp., New York, NY, USA) with a 95% confidence interval (CI). Two-tailed *p*-values < 0.05, were considered statistically significant.

## 3. Results

### 3.1. Infection Status of MAP

The overall prevalence of MAP in sheep was 3.34% (53/1585). Among the 12 prefectures and cities, the prevalence of MAP ranged from 0% (0/100) to 7.73% (15/194). In the eastern, central, and western regions, the overall prevalence rates were 4.74% (31/654), 3.55% (14/394), and 1.49% (8/537), respectively. Statistical analysis showed no significant difference between the eastern and central regions (odds ratio [OR] = 1.351, 95% CI: 0.709–2.571, *p* = 0.432). However, a significant difference was observed between the eastern and western regions (OR = 3.290, 95% CI: 1.500–7.220, *p* = 0.002) and between the central and western regions (OR = 2.436, 95% CI: 1.012–5.865, *p* = 0.049) (Table 1).

The prevalence of MAP in intensively farmed sheep was 3.56% (31/903), compared with 3.23% (22/682) in free-range sheep. No significant difference was observed between the two groups, with an OR of 0.938 (95% CI: 0.538–1.634, *p* = 0.888). In contrast, the prevalence of MAP in sheep (3.79%, 51/1346) was significantly higher than that in goats (0.84%, 2/239), with an OR of 4.667 (95% CI: 1.128–19.299, *p* = 0.017) (Table 1).

### 3.2. Genotype Identification MAP

The overall prevalence rates of C- and S-type MAP were 2.90% (46/1585) and 0.44% (7/1585), respectively, with a significant difference (OR = 6.738, 95% CI: 3.033–14.970, *p* < 0.01). The prevalence rates of C-type MAP in the eastern, central, and western regions were 4.43% (29/654), 1.08% (9/384), and 1.49% (8/537), respectively. There was no significant difference in the prevalence of C-type MAP between the eastern and central regions, nor between the central and western regions. However, the difference between the eastern and western regions was highly significant, with an OR of 3.068 (95% CI: 1.391–6.769, *p* = 0.004). The prevalence rates of S-type MAP in the eastern, central, and western regions were 0.31% (2/654), 1.30% (5/384), and 0% (0/537), respectively. No significant differences were observed in the prevalence of C-type MAP between the eastern and central regions or between the eastern and western regions. However, there was a significant difference between the central and western regions (*p* = 0.012). The prevalence rates of C-type MAP in sheep and goats were 3.27% (44/1346) and 0.84% (2/239), respectively, with a significant difference (OR = 4.005, 95% CI: 0.964–16.631, *p* = 0.036). In contrast, the prevalence rates of S-type MAP in sheep and goats were 3.27% (7/1346) and 0% (0/239), respectively, without significant difference (*p* = 0.603). For C-type MAP, the prevalence in intensively farmed sheep was 3.10% (28/903), compared with 2.64% (18/682) in free-range sheep, without significant difference (OR = 1.084, 95% CI: 0.595–1.978, *p* = 0.879). Similarly, for S-type MAP, the prevalence in intensively farmed sheep was 0.33% (3/903), compared with 0.59% (4/682) in free-range sheep, without significant difference (OR = 0.565, 95% CI: 0.126–2.533, *p* = 0.472) (Table 1).

## 4. Discussion

PTB was first described in the 19th century by Johne and Frothingham [18], and its causative agent was officially named *Mycobacterium avium* subsp. *paratuberculosis* in 1923 [19]. In China, the first occurrence of bovine PTB was reported in Inner Mongolia in 1953 [20], whereas that of ovine PTB was reported in Inner Mongolia and Jilin in 1971. Subsequently, PTB has occurred in many different provinces and cities in China [15]. Animals infected by MAP, whether clinically or subclinically affected, can shed live MAP in both their feces and milk [21]. Persistent environmental contamination by MAP has led to the spread of PTB, resulting in considerable economic losses in numerous regions worldwide [22]. At present, PTB is a major global concern in the field of animal health [23] and is classified as a “neglected disease” [24]. No country has claimed to be free from MAP infection [22]. However, underreporting and underestimation of MAP prevalence are widespread issues in many countries [2]. Effective control of PTB requires a comprehensive understanding of the regional prevalence of PTB and the distribution of MAP subtypes.

As shown by this survey of MAP in sheep feces rectally collected across the entire Inner Mongolia, the overall prevalence was 3.34% (53/1585). The low level of individual prevalence observed was mainly caused by MAP that can be intermittently shed [17] and the fecal shedding and end time after infection. In experimental challenge models, fecal shedding occurs 14 days post-inoculation [25]. In a previous study, fecal samples collected 42 days post-exposure in a caprine model were positive for MAP [26]. There are also reports that transient shedding began after 2 months post-inoculation [27]. A long-term study documented intermittent shedding in 10 sheep during the first year [28]. Of course, the worsening of this infection may lead to continuous daily shedding. Alternatively, shedding may cease permanently within 16 months post-exposure [29]. As regards the herd-level prevalence of bovine PTB, a few countries, such as Thailand, Norway, and Sweden, have been classified as having a low prevalence (<1%), whereas the prevalence of other countries, such as Belgium, Canada, Chile, Denmark, France, Germany, India, Israel, Italy, The Netherlands, New Zealand, Panama, Republic of Ireland, Spain, UK, USA, and Uruguay, exceeded 20% [2]. Pathogen-based studies have even reported herd-level prevalence rates of up to 70% in the USA in 2007 [30] and 46% in Canada [31]. In China, the seroprevalence rates of bovine PTB in Shandong Province in 2011 and 2012 were 27.96% and 14.93% [32]. In Tibet, Shanghai, and Guangxi Provinces, the seroprevalence rate of bovine PTB varied between 2% and 4%, whereas that in Inner Mongolia varied between 0% and 73.4% [33]. The seroprevalence rates of ovine PTB across different continents were as follows: 14.66% in Asia, 4.79% in Europe, 24.45% in South America, 1.34% in Africa, and 5.38% in North America [13]. In this study, the prevalence is relatively close to those in Europe and North America but lower than those in Asia and South America. In addition, this study found that the prevalence of MAP in the eastern and central regions of Inner Mongolia was significantly higher than that in the western region. These findings suggest that geographic factors, including longitude and latitude, as well as climatic conditions, may play a pivotal role in influencing the spread of MAP. However, significant variations in the prevalence of PTB persist among different countries within the same continent and across different time periods within the same country [13]. Therefore, identifying the risk factors for MAP transmission and ranking their importance are complex issues requiring extensive research for further confirmation. Notably, in studies focusing on chronic diseases, such as PTB, including the present study, a significant limitation is the neglect of age stratification in sample collection. This oversight is a potential major contributing factor to the discrepancies in the reported prevalence. Furthermore, this study found that the fecal prevalence of MAP in sheep was significantly higher than that in goats, which contrasts with literature reports indicating higher seroprevalence in goats than in sheep [34]. Therefore, further research is warranted to determine whether goats or sheep play a more pivotal role in the transmission and maintenance of MAP. Moreover, the farming method is generally regarded as a crucial factor influencing the spread of pathogenic bacteria, and the main MAP transmission route is precisely the fecal–oral route [35]. MAP has the ability to survive for extended periods of time in the environment: 152–246 days in pastures and up to 6–18 months in water [36]. The main risk factor potentially associated with MAP exposure is the absence of perimeter livestock fencing [34]. The results of this study suggest that there is no significant difference in MAP prevalence between intensively farmed and free-range sheep. Therefore, systematic research is warranted to identify the primary risk factors for MAP transmission, including the broader environmental exposure of free-range sheep, the higher stocking density in intensive farming, and the long-term fixed breeding environments.

In this study, the overall prevalence of C-type MAP (2.90%, 46/1585) was significantly higher than that of S-type MAP (0.44%, 7/1585), indicating that sheep in Inner Mongolia are predominantly infected with C-type MAP. The prevalence of C-type MAP significantly varied between regions: 4.43% (29/654) in the eastern region and 1.49% (8/537) in the western region. Similarly, the prevalence of S-type MAP significantly differed between regions: 1.30% (5/384) in the central region and 0% (0/537) in the western region. In addition, the prevalence of C-type MAP was significantly higher in sheep (3.27%, 44/1346) than in goats (0.84%, 2/239). This is inconsistent with literature reports indicating that ovine PTB is predominantly caused by S-type MAP and caprine PTB is predominantly caused by C-type MAP [7]. Our laboratory has also previously discovered that both sheep and goats in Inner Mongolia can be infected with these two types of MAP [15]. There are epidemiological trends associated with Type C and S strains with respect to transmission, host preference, and susceptibility to infection [37]. Countries such as Canada, New Zealand, the Faroe Islands, South Africa, Morocco, and Australia have reported S-type MAP infections in sheep. In Spain, sheep were predominantly infected with Type S strains [38]. Reports from the UK indicated that sheep were commonly infected with Type C strains [39]. In India, both sheep and goats were exclusively infected with Type C strains [40]. The literature shows that Type S strains are predominantly associated with sheep, whereas Type C strains are commonly isolated from cattle but exhibit a broad host range [5]. Therefore, neither Type S nor Type C MAP strains exhibit strict host specificity, and both sheep and goats can be infected by these two subtypes. In addition, reports suggest that the virulence of Type S and C strains may differ among different host species [41]. We need to conduct in-depth research on the host preference and virulence of MAP.

It is well established that the prevalence of PTB is considerably influenced by detection materials and diagnostic methods. PTB can be categorized into four infection stages: silent infection, subclinical disease, clinical stage, and advanced clinical stage [42]. In the first two stages, the bacterial load is typically low, and intermittent shedding of the pathogen occurs [43]. In fact, the outcome of exposure to PTB is also related to host-related characteristics, such as age at exposure and breed, and pathogen-related factors, such as MAP dose, strain, and type [29,44]. Permanent shedding cessation may occur 16 months post-challenge [29]. Therefore, PTB detection and diagnosis are particularly challenging. At present, the gold standard for MAP diagnosis is culture and identification [45]; however, this method requires considerable time. Furthermore, serological tests for detecting anti-MAP antibodies may exhibit delayed response owing to the prolonged interval of the humoral immune response in infected sheep [46]. The PCR method can be used to directly detect transient MAP shedding in feces [17]. A more sensitive qPCR method can effectively distinguish between low and high shedders [28]. qPCR using the SYBR green technology was employed to directly detect MAP in feces [47]. However, its specificity was determined by the primers, and the possibility of amplifying nonspecific sequences remained [48]. qPCR based on probe chemistry exhibits greater sensitivity and specificity than the other methods [16]. The qPCR method employed in this study is time-efficient, is easy to perform, and yields highly accurate results. Moreover, it not only identified MAP but also distinguished between Types S and C through a two-step process [17]. In this study, a two-step method was employed. A MAP-specific qPCR with the use of the primer pair 7132, which targets a DNA segregation ATPase protein, is capable of detecting all MAP strains. A strain-specific qPCR using the Atsa primer pair, which targets the arylsulfatase gene, effectively differentiates between Types S and C. This method also demonstrates higher sensitivity than the previously employed conventional methods [17]. Although the two-step method has several advantages, it also has certain limitations. Specifically, in the second step of detection, it can determine the presence of Type S MAP but cannot rule out the possibility of co-infection with S-type and C-type MAP. By introducing an additional pair of specific probes for Type C MAP, the two-step method can be expanded into a three-step approach to accurately identify potential mixed infections. Additionally, the method employed in this study lacks the resolution to further differentiate Type S into its sub-group Types I and Ⅲ. Actually, polymorphisms in *gyrA* and *gyrB* genes have been used early on to distinguish MAP Type I, II, and III Isolates [49], and the theory and method have been confirmed and validated in subsequent research [50,51]. The latest literature also emphasizes the critical role of *gyrA* and *gyrB* genes in the typing of Types I and III [52]. Moreover, the recently discovered variants in *gyrA* and *gyrB* genes do not interfere with the current typing of Type I and Type III lineages [53]. In future research, a qPCR method can be designed to differentiate among MAP Type I, II, III Isolates by using *gyrA* and *gyrB* genes, as well as to identify mixed strain infections.

## 5. Conclusions

This study conducted a comprehensive molecular epidemiological investigation of sheep across the entire Inner Mongolia region, revealing an overall prevalence of MAP in sheep of 3.34% (53/1585). The prevalence of MAP varied among the 12 leagues and cities, ranging from 0% (0/100) to 7.73% (15/194). Significant differences in prevalence were observed between the eastern and western regions, the central and western regions, intensively farmed and free-range sheep, and between sheep and goats. The overall prevalence of C-type MAP and S-type MAP was 2.90% (46/1585) and 0.44% (7/1585), respectively, indicating that both MAP subtypes coexist in the eastern and central regions of Inner Mongolia. These findings provide a critical basis for the prevention and control of PTB in the region. We suggest restricting the movement of infected flock within the country, culling infected sheep, and sterilizing the rearing environment.

## Figures and Tables

**Table 1:** Prevalence of MAP and assemblages, as determined using the breed, feeding method, and regions.

Factor			Sample Size			No. Positive (%) (95% CI)	*p* Value	OR (95% CI)	C Type			S Type		
									No. Positive (%) (95% CI)	*p* Value	OR (95% CI)	No. Positive (%) (95% CI)	*p* Value	OR (95% CI)
Breed	Sheep		1346			51 (3.79%) (2.77–4.81)	0.017	4.667 (1.128–l19.299)	44 (3.27%) (2.32–4.22)	0.036	4.005 (0.964–16.631)	7 (0.52%) (0.14–0.90)	0.603	–
	Goat		239			2 (0.84%) (−0.33–2.00)			2 (0.84%) (−0.33–2.00)			0		
Feeding method	Intensive		903			31 (3.56%) (2.24–4.62)	0.888	0.938 (0.538–1.634)	28 (3.10%) (1.97–4.24)	0.879	1.084 (0.595–1.978)	3 (0.33%) (0.04–0.71)	0.472	0.565 (0.126–2.533)
	Free range		682			22 (3.23%) (1.90–4.56)			18 (2.64%) (1.43–3.85)			4 (0.59%) (0.01–1.16)		
Regions	Eastern region	Hinggan League	194	15 (7.73%)	654	31 (4.74%) ^a^ (3.11–6.37)	0.432	1.351 (0.709–2.571)	29 (4.43%) ^a^ (2.85–6.02)	0.087	1.985 (0.930–4.238)	2 (0.31%) ^ab^ (−0.12–0.73)	0.111	0.239 (0.046–1.236)
		Hulun Buir	160	4 (2.50%)										
		Chifeng	100	0 (0%)										
		Tongliao	200	12 (6.00%)										
	Central region	Hohhot	184	3 (1.63%)	394	14 (3.55%) ^ab^ (1.72–5.39)	0.002	3.290 (1.500–7.220)	9 (1.08%) ^ab^ (0.80–3.77)	0.004	3.068 (1.391–6.769)	5 (1.30%) ^b^ (0.16–2.38)	0.504	–
		Xilingol League	100	4 (4.00%)										
		Ulanqab	110	7 (6.36%)										
	Western region	Alxa League	98	0 (0%)	537	8 (1.49%) ^b^ (0.46–2.52)	0.049	2.436 (1.012–5.865)	8 (1.49%) ^b^ (0.46–2.52)	0.459	1.546 (0.591–4.043)	0 ^a^	0.012	–
		Ordos	100	2 (2.00%)										
		Baotou	100	0 (0%)										
		Wuhai	84	1 (1.19%)										
		Bayannur	155	5 (3.22%)										
Total			1585	3.34% (53/1585)					2.90% (46/1585)			0.44% (7/1585)		

Superscript a, b indicates that the two different categories were significantly different. The superscript a, a indicates that the two different categories were not significantly different.

## Data Availability

The original contributions presented in this study are included in the article. Further inquiries can be directed to the corresponding authors.

## Abbreviations

PTB: Paratuberculosis; MAP: *Mycobacterium avium* subsp. *paratuberculosis*; STHS: Small-Tail Han sheep; PCR: polymerase chain reaction; OTUs: operational taxonomic units.
